# Establishment of a Pilot Newborn Screening Program for Spinal Muscular Atrophy in Saint Petersburg †

**DOI:** 10.3390/ijns10010009

**Published:** 2024-01-25

**Authors:** Anton Kiselev, Marianna Maretina, Sofia Shtykalova, Haya Al-Hilal, Natalia Maslyanyuk, Mariya Plokhih, Elena Serebryakova, Marina Frolova, Natalia Shved, Nadezhda Krylova, Arina Il’ina, Svetlana Freund, Natalia Osinovskaya, Iskender Sultanov, Anna Egorova, Anastasia Lobenskaya, Alexander Koroteev, Irina Sosnina, Yulia Gorelik, Olesya Bespalova, Vladislav Baranov, Igor Kogan, Andrey Glotov

**Affiliations:** 1 Department of Genomic Medicine Named after V.S. Baranov, D.O. Ott Research Institute of Obstetrics, Gynecology and Reproductology, Mendeleevskaya Line 3, 199034 Saint Petersburg, Russia; marianna0204@gmail.com (M.M.); sofia.shtykalova@gmail.com (S.S.); hayahilal.992@gmail.com (H.A.-H.); nmasl@yandex.ru (N.M.); maria86pola2007@list.ru (M.P.); el.a.serebryakova@mail.ru (E.S.); natashved@mail.ru (N.S.); nadushka.5@mail.ru (N.K.); arina-ilina-23@yandex.ru (A.I.); svetafreundmax@gmail.com (S.F.); timbuctu@mail.ru (I.S.); egorova_anna@yahoo.com (A.E.); shiggerra@mail.ru (O.B.); ikogan@mail.ru (I.K.); anglotov@mail.ru (A.G.); 2 Saint Petersburg State Medical Diagnostic Center (Genetic Medical Center), Tobolskaya Street 5, 353912 Saint Petersburg, Russia; mom1988@yandex.ru (M.F.); lobenskaya@mail.ru (A.L.); alexkoroteev@mail.ru (A.K.); 3 Saint Petersburg State Budgetary Healthcare Institution “Consulting and Diagnostic Center for Children”, Aleksa Dundić Street 36/2, 192289 Saint Petersburg, Russia; sosnina_kdcd@mail.ru; 4 Children’s City Multidisciplinary Clinical Specialized Center of High Medical Technologies, Avangardnaya Street 14, 198205 Saint Petersburg, Russia; gorelikjv@mail.ru

**Keywords:** spinal muscular atrophy, newborn screening, *SMN1*, *SMN2*, real-time PCR, dried blood spots, SMA incidence, carrier frequency

## Abstract

Spinal muscular atrophy 5q (SMA) is one of the most common neuromuscular inherited diseases and is the most common genetic cause of infant mortality. SMA is associated with homozygous deletion of exon 7 in the *SMN1* gene. Recently developed drugs can improve the motor functions of infants with SMA when they are treated in the pre-symptomatic stage. With aim of providing an early diagnosis, newborn screening (NBS) for SMA using a real-time PCR assay with dried blood spots (DBS) was performed from January 2022 through November 2022 in Saint Petersburg, which is a representative Russian megapolis. Here, 36,140 newborns were screened by the GenomeX real-time PCR-based screening test, and three genotypes were identified: homozygous deletion carriers (4 newborns), heterozygous carriers (772 newborns), and wild-type individuals (35,364 newborns). The disease status of all four newborns that screened positive for the homozygous *SMN1* deletion was confirmed by alternate methods. Two of the newborns had two copies of *SMN2*, and two of the newborns had three copies. We determined the incidence of spinal muscular atrophy in Saint Petersburg to be 1 in 9035 and the SMA carrier frequency to be 1 in 47. In conclusion, providing timely information regarding *SMN1*, confirmation of disease status, and *SMN2* copy number as part of the SMA newborn-screening algorithm can significantly improve clinical follow-up, testing of family members, and treatment of patients with SMA.

## 1. Introduction

Spinal muscular atrophy is a severe autosomal recessive disorder characterized by progressive muscular weakness caused by degeneration of motor neurons of the spinal cord. SMA is the most common genetic cause of infant mortality, with an incidence of about 1 in 6000–10,000 live births and a carrier frequency of 1:40–1:60 [1,2]. SMA is classified into four main clinical groups (types I-IV) based on the age of onset and severity of symptoms [3,4]. SMA Type I (Werdnig-Hoffmann disease), the most acute form, is characterized by onset in the first six months of life, failure to achieve sitting, and death within the first two years of life, often caused by respiratory distress. Patients with SMA type II present with their first symptoms after the age of six months, survive beyond two years, and can sit unsupported, but never gain the ability to walk. The milder manifestation, SMA type III (Kugelberg–Welander disease), develops after 18 months of life; these patients achieve the ability to walk and have life expectancies close to that of the healthy population. Patients with SMA type IV usually present with their first symptoms in the second or third decade of life and have normal life expectancies [3].

SMA is caused by homozygous mutations in the *SMN1* gene [5,6]. About 97% of patients have homozygous deletions of the *SMN1* gene; the rest have small intragenic mutations [7]. The *SMN1* gene has a nearly identical copy—the *SMN2* gene—that is the result of duplication and inversion of a chromosomal segment of around 500 kb in chromosome 5q13. *SMN1* differs from *SMN2* by several single-nucleotide changes [5]. Only one difference is functionally important—a translationally silent transition c.840C>T at position +6 in exon 7 that weakens the exonic splice site, causing exclusion of exon 7 from most *SMN2* transcripts and production of a truncated, non-functional SMN protein [8,9]. A small amount of full-length SMN protein is still produced by the *SMN2* gene, making this gene a principal modifier of disease severity in SMA patients. There is now growing evidence that additional factors contributing to SMA severity can be found among the multiple proteins interacting with SMN or affecting motor neuron survival, in epigenetic modifications, and among the transcriptional or splicing factors influencing *SMN2* expression [10,11,12,13,14]. In rare cases, protective modifiers may substantially ameliorate SMA progression, with effects as significant as the almost complete absence of symptoms [15,16]. Nonetheless, only pathogenetic treatment really has the potential to change the course of the disease. Such treatments are especially effective when they are given before symptoms arise.

Antisense oligonucleotide technology has been successfully used to modulate inclusion of *SMN2* exon 7 and has potential as a therapy for SMA [17]. Nusinersen was the first drug approved to treat spinal muscular atrophy [18]. It was registered in December 2016 as an orphan drug for SMA by the US Food and Drug Administration and has been available in Russia since 2019. Another *SMN2* drug that modifies pre-mRNA splicing, risdiplam, was registered in Russia in 2020 [19]. Risdiplam is an orally administered drug that increases production of full-length *SMN2* mRNA and spreads into the central nervous system (CNS), as well as the peripheral tissues [20].

Gene-replacement therapies are highly attractive therapeutic approaches for the treatment of spinal muscular atrophy, as SMA is classified as a monogenic disease. Many studies have found that injection of self-complementary scAAV9 vectors expressing full-length human SMN protein in various mouse models of severe SMA significantly increased their lifespan and reduced disease severity [21,22]. The Dallas biotech company AveXis has gained fast-track FDA approval for its scAAV9.CB.SMN vector, which Foust et al. found crosses the blood-brain barrier [23]. Onasemnogeneabeparvovec, known as AVXS-101 and sold commercially as Zolgensma, is a new treatment for SMA. It is a biologic gene-therapy drug that was approved in Russia in 2021. It consists of self-complementary AVV9 virus capsids that contain an *SMN1* transgene. It is believed that a single dose of the drug might have a lasting effect throughout the lifetime of the patient.

Importantly, the efficacy of any SMA therapy is highly dependent on the time when treatment is initiated. Considering that the goal of existing treatments is to stop disease progression, rather than to reverse motor-neuron death, therapeutic interventions should begin before the onset of symptoms. The importance of SMN as a protein with a housekeeping role and motoneuron-specific functions accounts for the narrow therapeutic window, especially in individuals with the most severe forms of SMA [24]. As most SMA cases develop in the early postnatal period, newborn screening for SMA is essential for diagnosis and timely administration of appropriate treatment. Evidence from in vivo studies in animal models and from clinical trials on children affected by SMA type I substantiate the idea that early therapeutic intervention is correlated with better motor performance and rescue of the pathological phenotype [25].

Currently, there is no newborn screening for spinal muscular atrophy in Saint Petersburg. With a population of over 5,200,000 and a birth rate of 52,000 newborns per year, Saint Petersburg is a representative Russian megapolis. Our main aim is the establishment of an newborn-screening program for SMA in Saint Petersburg. The second aim of this study is to determine the incidence of SMA and the carrier frequency in the population of Saint Petersburg.

## 2. Materials and Methods

### 2.1. Study Recruitment

Twenty-one Saint Petersburg maternity hospitals participated in the pilot SMA screening program. Each hospital provided mothers with informed-consent forms and informational brochures. Dried blood spots (DBSs) were taken for mandated neonatal screening, and the signed consent forms and corresponding DBSs were transported to the Genetic Medical Center. Only the DBSs from infants whose mothers had provided consent for this test were transported to the Genetic Medical Center. The SMA test was provided at no cost to participants.

### 2.2. Assay Validation

Validation of the GenomeX technology proposed for use in the SMA screening analysis was performed on DNA samples with known genotypes extracted from whole blood by means of phenol-chloroform extraction. A total of 250 samples were analyzed: *SMN1* exon 7 homozygous deletion carriers (*N* = 50), heterozygous carriers (*N* = 58) and wild-type individuals (*N* = 142). *SMN1* deletions and copy number were determined before validation with TaqMan real-time PCR technology, as reported previously [26].

A trial screening test was performed using the GenomeX assay kit (Genome-Mix LLC, Moscow, Russia) on 400 DBS punches with unknown *SMN1* copy number that were provided by the Genetic Medical Center.

### 2.3. Specimens

This study was performed using 250 DNA samples collected in large-scale research facilities #3076082, “Human Reproductive Health”, in the D.O. Ott Research Institute of Obstetrics, Gynecology and Reproductology.

Four hundred DBSs for the validation stage and 36,140 DBSs for the pilot screening stage were obtained from the Genetic Medical Center. Filter-paper blood spots were obtained by heel puncture on the 4th day after birth (or on the 7th day for premature babies).

Capillary blood samples from SMA heterozygous carriers and SMA homozygous deletion carriers were obtained for verification from the Genetic Medical Center. DNA was extracted by means of salt (NaCl) extraction [27].

### 2.4. SMA Screening Assay

#### 2.4.1. DNA Extraction

1. A 1× GenomeX elution reagent was prepared by diluting the 100× solution with ampouled or bidistilled water 1:99 to obtain 250 µL of 1× solution per sample.
2. A 3.2 mm diameter circle was punched out from a DBS card into a 0.2 mL tube or a 96-well plate using a puncher.
3. 100 µL of 1× elution reagent was added to each tube.
4. Next, the tubes were incubated for 15 min at room temperature.
5. The liquid was stirred by pipetting twice and then removed from the tube.
6. Steps 3–5 were repeated one more time.
7. 50 mL of 1× elution reagent was added to the washed filter.
8. The tubes/plated were closed and centrifuged to remove droplets from the lids.
9. The samples were heated for 15 min at +99 °C in a hot-lid Bio-Rad T100 thermocycler.
10. The samples were removed to +4 °C and stored at that temperature for up to 3 weeks.

#### 2.4.2. Performing Real-Time PCR

1. GenomeX MasterMix was defrosted and added to the sample, along with polymerase (17.6 µL of MasterMix + 0.4 µL of polymerase per sample). Each DNA sample was analyzed twice.
2. 18 µL of prepared mix was added to each well of a 96-well plate.
3. 2 µL of DNA sample was added to each well. No DNA was added to 2 of the wells (the contamination controls).
4. The plate was centrifuged to remove droplets from the lid.
5. The plate was placed in a real-time PCR device under the following conditions: preincubation, 95 °C for 5 min, then 40 cycles of 94 °C for 15 s and 62 °C for 1 min.

Several real-time PCR machines were used: QuantStudio 5 (Applied Biosystems, Carlsbad, CA, USA), LightCycler 96 (Roche, Basel, Switzerland), Applied Biosystems 7500 (Applied Biosystems, Thermo Fisher Scientific, Waltham, MA, USA), and Rotor-Gene 3000 (Corbett Research, Mortlake, NSW, Australia).

The fluorescent signal was detected on the FAM/Sybr Green and ROX/Texas Red channels.

#### 2.4.3. Analysis of the Results

The results were obtained via ΔΔCt/relative quantitative analysis. When a run was performed on the Rotor-Gene 3000, a calibration curve was prepared to calculate the results. A sample with 2 copies of the *SMN1* gene was used as a calibrator; the value for this sample was set to 1 and corresponded to the relative amount of the *SMN1* gene according to the formula SMN1 = [SMN1]/[reference], where [SMN1] is the concentration of the real-time PCR product from the *SMN1* gene (FAM channel). Here, [reference] is the concentration of the real-time PCR product from the reference gene (ROX channel. Thus, a value of 0 for a sample corresponded to an SMA patient, a value of 0.5 to an SMA carrier (1 copy of *SMN1*), a value of 1 to 2 *SMN1* copies, a value of 1.5 to 3, etc. Ct on the ROX detection channel should be <33.

### 2.5. Screening, Notification of Results and Confirmatory Testing

When *SMN1* deletions were found, family members were invited to the Genetic Medical Center for medical genetic counseling and subsequent blood sampling (the whole family in the case of homozygous deletion and a newborn in the case of heterozygous deletion). Family members received printouts with the results of the SMA neonatal screening on the DBS only when *SMN1* deletions were found. *SMN1* deletions were verified in the D.O. Ott Research Institute using DNA samples extracted from capillary blood. In the case of homozygous deletion, MLPA analysis was performed according to the manufacturer’s instructions (SALSA MLPA Probemix P021 SMA, MRC-Holland, Amsterdam, The Netherlands). MLPA results were visualized on the Applied Biosystems 3130 Genetic Analyzer (Thermo Fisher Scientific, Waltham, MA, USA). After verification, family members received another printout with results based on the analysis performed on DNA extracted from whole blood and received genetic counseling.

## 3. Results

### 3.1. Validation Study

We evaluated the Genome X real-time PCR-based assay using previously analyzed samples with different genotypes: homozygous *SMN1* exon 7 deletion carriers, heterozygous carriers and wild-type individuals. We found clearly defined, non-overlapping ranges of values in the *SMN1* copy number between the samples with 0, 1, and 2 or more copies of *SMN1* exon 7, regardless of the *SMN2* copy number. The significance of the differences was confirmed by analysis of variance (*p* < 0.001). Based on these data, ranges of *SMN1* copy number values were assigned to each *SMN1* genotype: a range of 0.23–0.599 was considered to correspond to heterozygous carriers, whereas a range of 0.79–1.23 was considered to correspond to individuals negative for the *SMN1* exon 7 deletion (2 *SMN1* copies), and a result of zero was considered to correspond to homozygous deletion carriers (Table 1). There was 100% concordance between the *SMN1* copy number determined by the Genome X assay and the known genotypes of 250 control samples. Specificity of the assay is thus 100%, and the sensitivity is also 100% if we do not take in account the inability of this real-time PCR-based method to detect point mutations in the *SMN1* gene. Otherwise, the sensitivity of the assay is 97%, based on the incidence of *SMN1* point mutations in compound heterozygous carriers [7].

Additionally, de-identified DBSs from newborns (*N* = 400) with unknown *SMN1* copy number were provided by the Genetic Medical Center for the analysis. The total number of DBS samples roughly corresponds to the number of babies who are born in Saint Petersburg every two days. A total of 48 h were spent on screening the 400 samples in the validation study. According to the results, 8 individuals (2%) were SMA carriers, having one copy of *SMN1* exon 7; 360 individuals (90%) have two copies of *SMN1*; and 32 (8%) have three copies. The carrier frequency was thus estimated to be 1:50, a result that is in concordance with the reported data [28]. None had a homozygous *SMN1* deletion, and no samples failed.

### 3.2. Flow of Patient Samples and Data 

**Figure 1:**
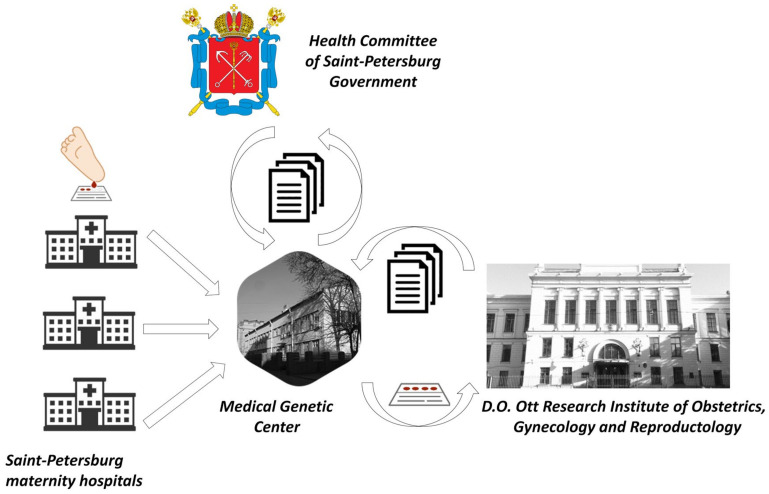
Overview of the flow of DBS and documentation for SMA newborn screening in Saint Petersburg.

For this study, no additional biological material from newborns was required because the residual blood spots collected for mandated NBS are sufficient for the SMA testing. The mandated DBS cards are collected under the order of the Health Committee of the Saint Petersburg Government, dated 05/29/2006, No. 220-r: “On mass screening of newborn children for hereditary diseases in Saint Petersburg”. According to this order, newborns are screened for five inherited diseases (phenylketonuria, cystic fibrosis, galactosemia, congenital adrenal hyperplasia, primary congenital hypothyroidism) in Saint Petersburg. DBS collection is carried out in maternity hospitals supervised by the Saint Petersburg Health Committee, as well as in federal institutions that include maternity hospitals, perinatal centers, and maternity wards. The final destination for the DBS cards was the Genetic Medical Center. Aliquots of samples (DBS punches) for SMA screening studies were taken daily (on weekdays) at agreed-upon hours in a specialized laboratory room of the Genetic Medical Center by its personnel under the supervision of D.O. Ott Research Institute research assistants. The mothers’ informed-consent forms and paper referrals for a SMA screening study were attached to the samples, which were then transported to the D.O. Ott Research Institute (Figure 1).

**Figure 2:**
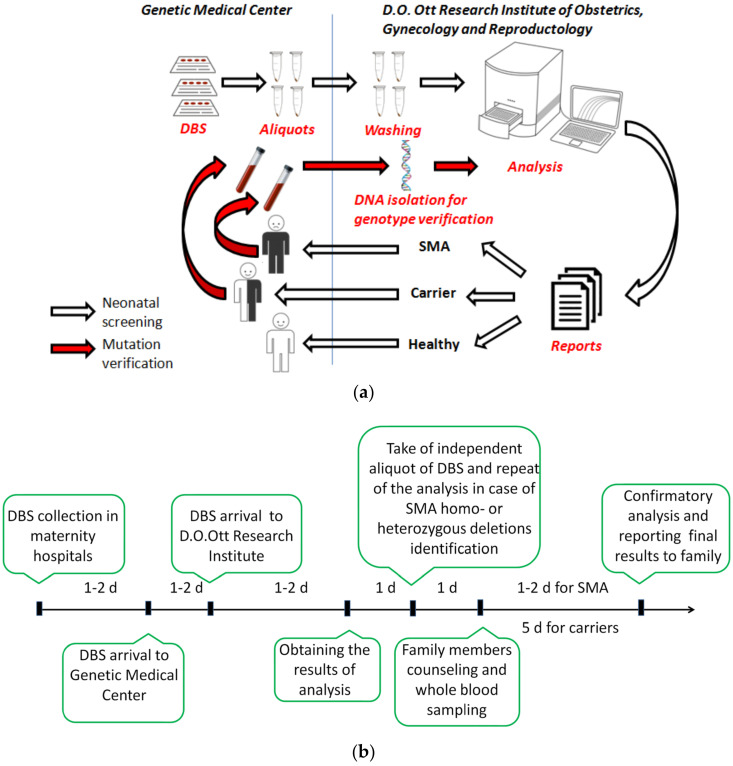
Screening algorithm (**a**) and workflow timeline (**b**) used in the SMA pilot study in Saint Petersburg.

Analysis at the D.O. Ott Research Institute was carried out within 3 working days of the arrival of the sample at the Genetic Medical Center. It took 1–2 days to transfer the DBSs to the Genetic Medical Center. If the screening results were positive (for presence of homozygous mutations in the *SMN1* gene) or the individual was identified as an SMA carrier, the independent aliquot of DBS was taken from the Genetic Medical Center and the analysis of the sample was repeated. If the results were then confirmed, the study coordinator sent a message to the Genetic Medical Center about the necessity for medical genetic consultation for the mother of a newborn who had an identified risk of developing SMA or who was a carrier for SMA. Quality control of the study was provided by a specialist external to this project who independently examined 10 previously analyzed random samples every week (blind control). In accordance with the screening algorithm, family members (the whole family in the case of homozygous deletion and a newborn in the case of a heterozygous deletion) were invited to the Genetic Medical Center for medical genetic counseling and subsequent blood sampling for SMA diagnosis or verification of carrier status in the D.O. Ott Research Institute (Figure 2a).

Verification of SMA diagnoses was carried out within 1–2 working days from the receipt of the whole-blood sample, while SMA carrier testing on blood samples was performed within 5–10 days (Figure 2b). Confirmatory testing in the case of a homozygous *SMN1* deletion always included determination of *SMN2* gene copy number due to the importance of *SMN2* as a disease modifier [15]. If the results of verification assay confirmed detection of homo- or heterozygous mutations in the *SMN1* gene, the mother of the newborn now known to be at risk of developing SMA or known to be a carrier of SMA were informed, and genetic counseling was provided by specialists of the Genetic Medical Center. If no mutations in the *SMN1* gene were found in the newborn, the mother was informed that there is no risk of developing SMA or of SMA carrier status. Also, positive results for SMA or SMA carrier status were simultaneously reported to neurologists in the Saint Petersburg Consulting and Diagnostic Center for Children.

### 3.3. Screening for SMA

Within the time period of the SMA pilot study, 36,140 of 36,217 consent forms for participation in the NBS program were received. Therefore, 99.79% of these parents agreed to SMA testing of their newborn children. This outstanding return rate is the result of the study awareness partially mediated by efforts of the Health Committee and the general public interest in the problem of SMA. The almost total agreement of parents to additional SMA testing differs markedly from the response rates of NBS programs in other countries [29,30].

In total, 36,140 newborns were screened, and three genotypes were detected among them. Our SMA NBS program resulted in the detection of four homozygous *SMN1* deletion carriers, 772 heterozygous carriers, and 35,364 wild-type individuals. No false-positive SMA cases were detected, and only two false-positive heterozygous carriers were found. The disease status of all four newborns who screened positive was confirmed by alternate methods—a different real-time PCR system and MLPA [26,31]. In all newborns with homozygous *SMN1* deletion, *SMN2* copy number was also determined. Two of these newborns had two copies of *SMN2*, and two had three copies. All the diagnosed individuals were found before the onset of symptoms, and all have already received gene therapy with onasemnogene abeparvovec.

### 3.4. Determination of the of Frequency SMA Carrier Status in Newborns

SMA is the leading genetic cause of infant mortality, and it is important to carry out studies on its prevalence and incidence. The establishment of an SMA NBS program by means of a GenomeX real-time PCR-based screening test gives us a unique opportunity to gain additional information on the incidence of SMA in Saint Petersburg and to determine the frequency of carrier status. Information on the possibility of obtaining information of SMA carrier status was included to the consent form, and the parents could choose not to be informed of their newborn’s status. However, none of the parents chose not to learn this information. Moreover, to date, most of the parents of newborns with heterozygous *SMN1* deletions have received medical genetic counseling. Some parents used this information for pre-conception SMA screening, and several dozen SMA carriers were found among them and their children. This work is underway and will be continued. It should be noted that no families with SMA risk were found among the parents of heterozygous newborns.

In conclusion, in the Saint Petersburg newborn population, we found the incidence of spinal muscular atrophy to be 1 in 9035 and the frequency of SMA carrier status to be 1 in 47. Our data are in full agreement with previously reported data on SMA incidence and carrier frequency [28]. However, the data differ from the international summarized data from NBS SMA programs and the data from a study of the carrier frequency in three populations in the Russian Federation [32,33].

## 4. Discussion

Here, we described the establishment and implementation of an exhaustive pilot newborn screening for spinal muscular atrophy in Saint Petersburg. This study enabled the detection not only of SMA patients, but of individuals who were exclusively SMA carriers, as well. The recently developed GenomeX assay kit (Genome-Mix LLC, Moscow, Russia) that provided the technological basis of this project was first validated on DNA samples with known genotypes. This approach has the same limitations as other technologies used in SMA NBS programs all over the world, like the inability to detect the point mutations in *SMN1* that account for about 2–5% of SMA cases. However, the GenomeX test has an undoubted advantage in the detection of SMA carrier status. The testing protocol, which includes verification of homozygous and heterozygous *SMN1* deletions, was designed to include several independent quality-control steps. To minimize the incidence of mistakes, samples that were found to have homo- or heterozygous mutations in the *SMN1* gene were firstly re-tested by real-time PCR from same aliquot of the DBS, then from an independent aliquot of the DBS; in the final step in confirming the results, the analysis was performed on DNA isolated from whole blood. Moreover, a blinded control step, performed each week, consisted of the analysis of 10 random samples by an independent researcher. To obtain complete information in the case of homozygous *SMN1* deletion, the whole family was tested for *SMN1* and *SMN2* copy numbers.

Finally, this study detected 4 cases of SMA per 36,140 newborns, a result in accordance with incidence rate of SMA detected in worldwide NBS programs. The known SMA prevalence (1 in 9035 neonates) is a little bit lower than the rate previously described in Russia (Moscow), as well as in Germany, Italy, and Latvia, but is higher than that detected by newborn screenings in Taiwan, the USA, Belgium, Australia, Canada, and China [30,32,34,35,36,37,38,39,40,41,42].

The accuracy of detection of *SMN1* homozygous deletion is strengthened by the corresponding number of individuals identified as having heterozygous mutations. The ability of the technology used in our SMA NBS program to reveal carrier status serves as additional verification step because of its sensitivity.

The identification of 772 heterozygous carriers of SMA will allow implementation of programs for effective prevention of SMA in these families in the future. In fact, the project became the basis for the transition to pre-conception screening with the further use of primary and secondary prevention tools: preimplantation and prenatal diagnostics. In terms of the limitations of the test, as for most tests used in diagnostic laboratories, this method cannot detect silent (2 + 0) carriers, a group that accounts for 3% to 8% of SMA carriers [43,44].

Each of the four SMA patients identified in the pilot newborn screening program were treated with onasemnogene abeparvovec. SMA patients treated in the presymptomatic stage are expected to have better motor development and quality of life and much lower disease costs compared to patients identified by their symptoms, as described previously [32,45].

In conclusion, the provision of timely information on *SMN1* and confirmation of disease status, along with *SMN2* copy number, as part of a SMA newborn-screening program can significantly improve clinical follow-up, testing of family members, and treatment of SMA patients. Presymptomatic SMA detected is the basis of maximum therapeutic effectiveness, especially for patients with SMA type I. Information about carrier status can also be a part of pre-conception care.

## Figures and Tables

**Table 1:** Results of validation of SMA detection method.

Genotype	Wt/Wt	Del 7/Wt	Del 7/Del 7	Sensitivity */ Specificity
**Del 7/Del 7**	0	0	50	100%/100%
**Del 7/Wt**	0	58	0	100%/100%
**Wt/Wt**	142	0	0	100%/100%

* Possible point mutations in the *SMN1* gene are not taken into account.

## Data Availability

The data are not publicly available due to restrictions of the subjects’ agreement.
